# Patients’ characteristics and six-month outcomes of patients with atrial fibrillation in Kenya: a retrospective observational cohort study from the National Cardiovascular Registry

**DOI:** 10.11604/pamj.2025.52.41.47854

**Published:** 2025-09-26

**Authors:** Salim Abdallah, Jasmit Shah, Anders Barasa, Felix Barasa, Benard Gitura, Mzee Ngunga

**Affiliations:** 1 Department of Medicine, Aga Khan University, Nairobi, Kenya,; 2 Brain and Mind Institute, Aga Khan University, Nairobi, Kenya,; 3 Heart Failure Lead, Amager Hvidovre Hospital, University of Copenhagen, Hvidovre, Denmark,; 4 Department of Cardiology, Moi Teaching and Referral Hospital, Eldoret, Kenya,; 5 Department of Cardiology, Kenyatta National Hospital, Nairobi, Kenya

**Keywords:** Atrial fibrillation, anticoagulation, warfarin, cardiovascular, valvular disease, Kenya

## Abstract

**Introduction:**

atrial fibrillation is increasingly diagnosed in Kenya due to the persistence of rheumatic heart disease and the rising burden of cardiovascular risk factors. We aim to describe the baseline, clinical, treatment characteristics, and six-month outcomes of patients diagnosed with atrial fibrillation in Kenya.

**Methods:**

a retrospective observational cohort study design was employed. Data were obtained from three Kenyan referral hospitals, including public and private institutions. Baseline and six-month data were collected. Depending on the type of variable, data were summarized descriptively.

**Results:**

two hundred forty participants were enrolled, with a median age of 59.0 (IQR: 42.0-75.8). Women made up 54.4% (n=123) of the cohort. The median body mass index was 24.8 kg/m^2^(IQR: 21.1-29.2), and 62.8% (n=142) of participants were hospitalized at enrollment. Non-valvular atrial fibrillation (AF) was the predominant type, accounting for 77.4% (n=175) of cases, with persistent AF being the most common subtype (60.5%, n=137). At baseline, 77% (n=174) of participants were on anticoagulation therapy. The proportion with high-risk HAS-BLED and CHA_2_DS_2_-VASc scores at baseline was 10 (4.4%) and 62 (28.8%), respectively. Hypertension was the most prevalent comorbidity, affecting 39.4% (n=89) of participants. Nearly half (48.6%) had a preserved left ventricular ejection fraction. At the six-month follow-up, all participants remained on anticoagulation therapy. Mortality occurred in 17.7% (n=40) of participants, with cardiovascular causes accounting for 45.0% of these deaths.

**Conclusion:**

the predominant type was non-valvular atrial fibrillation. Enhancing screening for comorbidities and adopting a holistic approach to atrial fibrillation care could lead to better patient outcomes in Kenya.

## Introduction

Atrial fibrillation (AF) is the most common sustained cardiac arrhythmia, characterized by disorganized electrical activity in the atria, leading to ineffective atrial contractions and an irregular ventricular response [1]. It can present in different forms, including paroxysmal, persistent, and permanent AF, which are differentiated by the duration and recurrence of arrhythmic episodes [2]. The condition is influenced by a variety of risk factors, including genetic predispositions, cardiovascular conditions such as hypertension and valvular heart disease, metabolic disorders like diabetes and obesity, thyroid dysfunction, and inflammatory processes [3].

Globally, AF is a significant contributor to morbidity and mortality, impacting around 37.6 million individuals worldwide. Projections suggest that the prevalence could increase by over 60% by 2050 [4]. The condition's incidence rises with age, with approximately one in four middle-aged adults expected to develop AF during their lifetime [5]. The economic impact is also considerable, with healthcare costs in the United States alone exceeding $750 million annually, much of which is associated with managing complications such as stroke and heart failure [6,7].

Evidence suggests that African populations with AF often present with more severe clinical manifestations compared to patients in high-income countries. This disparity is believed to be driven by the high prevalence of rheumatic mitral disease and poor adherence to clinical practice guidelines, leading to suboptimal AF management [8,9]. According to Linz *et al*. the prevalence of AF in sub-Saharan Africa is estimated at 659.8 per 100,000 among men and 438.1 per 100,000 among women, underscoring a notable gender difference [10].

Despite the magnitude of the prevalence of AF in sub-Saharan Africa, limited data exist on patients´ characteristics, treatment received, and post-discharge follow-up, particularly in Kenya. This study aims to describe the baseline demographic, clinical profiles, and six-month follow-up characteristics of patients diagnosed with atrial fibrillation in Kenya. The research question guiding this study is: What are the demographic, clinical, and treatment characteristics, along with short-term outcomes, of patients diagnosed with atrial fibrillation in Kenya?

## Methods

**Study design and setting:** the study used data from the Kenya Heart Registry, an investigator-initiated research on cardiovascular diseases in Kenya, collected using a retrospective observational study design between December 2019 and December 2023. This is a multicenter heart registry aimed at understanding the epidemiology and outcomes of primary cardiovascular conditions, including heart failure, acute coronary syndromes, atrial fibrillation, and pulmonary thromboembolism across the country. This registry encompasses data from three leading national referral hospitals- Kenyatta National Hospital, Aga Khan University Hospital, and Moi Teaching and Referral Hospital- which serve as key centers for cardiovascular care in Kenya, catering to diverse urban and semi-urban populations.

**Study population and sampling:** the study included all adult patients diagnosed with AF during the study period. The inclusion criteria were as follows: 1) Electrocardiogram (ECG) evidence of AF not associated with recent cardiac surgery (within the past 2 weeks) or acute coronary syndrome (within the past 48 hours); 2) AF detected via Holter monitor; 3) AF episodes lasting more than 30 seconds identified by loop recorder; and 4) AF episodes lasting more than 30 seconds detected by cardiac pacemaker, implantable cardioverter-defibrillator (ICD), or cardiac resynchronization therapy (CRT) device.

No sampling technique was employed, as all eligible patient records from the three participating national referral hospitals were included in the analysis. The study utilized registry data and included all patient records that met the predefined eligibility criteria; therefore, no formal sample size calculation was conducted. Similarly, no power calculation was performed, as the study was descriptive and based on available data.

**Data collection:** data were collected using a standardized paper-based case report form, which captured a range of variables in patient demographics, clinical characteristics, treatment regimens, and outcomes at six months post-discharge. Once the case report forms were completed, the data were entered into a password-protected REDCap registry database with validation checks hosted at Aga Khan University, Nairobi, to limit data errors during entry. Data were collected immediately after, during, and six months post-admission. Six months after discharge, information was collected through telephone calls and follow-up on critical outcomes, including all pre-hospitalization causes, mortality, and anticoagulant utilization. Data on mortality and hospitalization were determined as reported by the participant or the participant's relatives during follow-up by telephone, review of discharge summaries, and death notification certificate. All these details were also entered into the registry database from which the data used in this study were extracted.

**Clinical outcomes:** the CHA_2_DS_2_-VASc score at baseline for each participant to assess stroke risk was calculated by assigning one point for each of the following characteristics: a history of congestive heart failure, hypertension, age ≥75 years, or diabetes mellitus, and 2 points are assigned for a history of stroke or transient ischemic attack [11-13]. The HAS-BLED bleeding risk score was calculated as a measure of baseline bleeding risk as the result of adding 1 point to hypertension, creatine ≥200 micromol/liter (1 point each), stroke, bleeding history or predisposition, labile INR, old age (≥65 years), and use of drugs (aspirin or clopidogrel) and alcohol concomitantly (1 point for each). Labile INR was quantified as 0 in every patient [14] based on the inclusion criteria at entry. Major bleeding was defined as a fall in hemoglobin level of at least 2gm/dL or the need for transfusion of 2 or more units of blood [15].

**Statistical analysis:** descriptive statistics, including frequency and percentages for categorical variables, summarized the study population’s demographic, clinical, and treatment characteristics and six-month outcomes, whilst the median (interquartile range) was reported for continuous variables. All analyses were conducted using R version 4.3.2 (2023-10-31 ucrt).

**Ethical considerations:** ethical approval for the study was obtained from the Aga Khan University Hospital institutional scientific ethics and review boards (Ref: 2019/REC-29 (v1)) and a research permit from the National Commission for Science, Innovation, and Technology. Written informed consent was obtained from all participants before their enrollment in the study. Patient confidentiality was maintained by anonymizing all data using a unique identifier. The study adhered to the principles of the Declaration of Helsinki, ensuring the protection of patient rights, privacy, and autonomy throughout the research process.

## Results

**Baseline characteristics of the participants:** the study included 226 participants, of whom 123 (54.4%) were females, with a median age in years of 59.0 (IQR: 42.0-75.8). The median body mass index was 24.8 kg/m^2^ (IQR: 21.1-29.2), with the majority classified in the normal weight category, followed by the overweight group and the underweight group, which was least represented. The persistent type of atrial fibrillation (AFIB) was the most predominant one (n=137; 60.6%). Participants with high CHA_2_DS_2_-VASc and HAS-BLED were 65 (28.8%) and 10 (4.4%), respectively. There were 175 (77.4%) of the patients with non-valvular disease, whilst the rest had valvular Atrial Fibrillation. One hundred thirty (78.3%) were classified as having a normal LA index, while those with a mild LA index were 20 (12.0%), and only eight each had a moderate and severe LA index. The most prevalent comorbidity was hypertension (n=89; 39.4%), followed by diabetes (n=29; 12.8%). There were only seven participants with a reported history of stroke/TIA. Data on RVSP were obtained from 183 participants with a median of 61.0 (IQR: 45.0-75.0), of whom 97 (53.0%) were classified as severely elevated (Table 1).

**Table 1:** baseline characteristics of the study participants, recruited from three teaching referral hospitals in Kenya, from December 2019 to December 2023 (N=226)

Characteristics	N = 226
**Gender, n (%)**	
Female	123 (54.4)
Median age (IQR), years	59.0 (42.0-75.8)
Body mass index (kg/m^2^), median (IQR)	24.8 (21.1-29.2)
**Categories of BMI, n (%)**	
Underweight	19 (8.8)
Normal	91 (42.3)
Overweight	64 (29.8)
Obese class	41 (19.1)
**Type of AFIB, n (%)**	
Paroxysmal	47 (20.8)
Persistent	137 (60.6)
Permanent	42 (18.6)
**CHA_2_DS_2_-VASc, n (%)**	
Low	121 (53.5)
Moderate	40 (17.7)
High	65 (28.8)
**HAS-BLED category, n (%)**	
Low risk	216 (95.6)
High risk	10 (4.4)
**AFIB disease, n (%)**	
Valvular atrial fibrillation	51 (22.6)
None-valvular disease	175 (77.4)
**LA index (n=166), n (%)**	
Normal	130 (78.3)
Mildly	20 (12.0)
Moderately	8 (4.8)
Severely	8 (4.8)
Hypertension, n (%)	89 (39.4)
Known CAD, n (%)	9 (4.0)
Diabetes, n (%)	29 (12.8)
Previous stroke/TIA, n (%)	7 (3.1)
RVSP (mmHg) (n=183), median (IQR)	61.0 (45.0-75.0)
**RVSP categories (n=183)**	
Normal	11 (6.0)
Mildly elevated	49 (26.8)
Moderately elevated	26 (14.2)
Severely elevated	97 (53.0)

BMI: body mass index; AFIB: atrial fibrillation; LA: left atrium

**Treatment characteristics:** the most common beta-blockers prescribed were carvedilol (41.1%), followed by bisoprolol (28.8%) and metoprolol (26.7%). Among the 174 participants on anticoagulation therapy, warfarin was the most frequently used anticoagulant by 93 (53.4%), followed by rivaroxaban (n=46; 26.4%), only three on dabigatran, and one on unfractionated heparin. Other anticoagulants included low-molecular-weight and unfractionated heparin (Table 2).

**Table 2:** medications prescribed to study participants, recruited from three teaching referral hospitals in Kenya, from December 2019 to December 2023 (N=226)

Medication	
**Beta blocker (n=146), n (%)**	
Atenolol	1 (0.7)
Propranolol	3 (2.1)
Carvedilol	60 (41.1)
Metoprolol	39 (26.7)
Bisoprolol	42 (28.8)
Labetalol	1 (0.7)
Digoxin (n=226), n (%)	83 (36.7)
**Anticoagulant (n=174), n (%)**	
Warfarin	93 (53.4)
Rivaroxaban	46 (26.4)
Dabigatran	3 (1.7)
Apixaban	16 (9.2)
Low molecular weight heparin	15 (8.6)
Unfractionated heparin	1 (0.6)

**Discharge characteristics:** there were only two in-hospital deaths, neither of which was unrelated to cardiovascular conditions. Additionally, two participants experienced intracranial or upper gastrointestinal bleeding, resulting in major bleeding. Of the participants, 174 (77.7%) were on anticoagulation therapy. Among them, 93 (53.5%) were on warfarin, 51 (29.3%) on rivaroxaban, 21 (12.1%) on apixaban, and nine on dabigatran. The median INR among warfarin users (n=93) was 1.4 (IQR: 1.2-1.7). Lastly, only 12 participants were on antiplatelet therapy, with seven on aspirin, four on clopidogrel, and one on ticagrelor (Table 3).

**Table 3:** in-hospital outcome of study participants, recruited from three teaching referral hospitals in Kenya, from December 2019 to December 2023 (N=226)

Variables	
Patients' status, Alive (n=226), n (%)	224 (99.1)
**Cause of death (n=2), n (%)**	
Non-cardiac cause	2 (13.3)
Bleeding complication (n=224), n (%)	2 (1.4)
HB-drop >2 g/dL (n=224), n (%)	2 (4.5)
Bleeding site: intracranial (n=224), n (%)	1 (0.7)
Bleeding site: upper GI (n=224), n (%)	1 (0.7)
On anticoagulation (n=224), n (%)	174 (77.7)
**Type of anticoagulation (n=174), n (%)**	
Warfarin	93 (53.5)
Rivaroxaban	51 (29.3)
Dabigatran	9 (5.2)
Apixaban	21 (12.1)
Warfarin last INR (n=93), median (IQR)	1.4 (1.2 - 1.7)
**Antiplatelet (n=226), n (%)**	
Aspirin	7 (3.1)
Clopidogrel	4 (1.8)
Ticagrelor	1 (0.6)

INR: International Normalized Ratio

**Six-month characteristics:** among the participants enrolled at baseline, 40 (17.7%) died within six months, with 27 (67.5%) passing away in the hospital. The most common cause of death was cardiac-related (n=18; 45.0%). Among those who survived, seven were hospitalized within six months, with five experiencing only one admission. Cardiac-related conditions remained the leading cause of hospitalization. Twelve participants (5.3%) underwent a repeat echocardiogram. The majority (n=7; 58.6%) had an ejection fraction (EF) of 50% - 70%, while only one had an EF of 40% - 49%. Regarding anticoagulation therapy, 142 participants (63.4%) were on anticoagulants. Among them, 74 (52.1%) were on warfarin, 45 (31.7%) on dabigatran, and only two on rivaroxaban. The median duration of warfarin use was 2.3 days (IQR: 2.0-2.6) (Table 4).

**Table 4:** patients' status at six months follow-up of study participants, recruited from three teaching referral hospitals in Kenya, from December 2019 to December 2023 (N=224)

Variables	
Patients' status, alive (n=224), n (%)	184 (82.1)
**Location of death (n=40), n (%)**	
Home	13 (32.5)
Hospital	27 (67.5)
**Cause of death (n=40), n (%)**	
Cardiac cause	18 (45.0)
Non-cardiac cause	6 (15.0)
Unknown	16 (40.0)
**Number of admissions (n=7), n (%)**	
1	5 (71.4)
2	1 (14.3)
3	1 (14.3)
**Cause of admission (n=7), n (%)**	
Cardiac cause	5 (71.4)
Non-cardiac cause	2 (28.6)
Repeat echo (n=224), n (%)	12 (5.3)
On anticoagulation (n=224), n (%)	142 (63.4)
**Type of anticoagulation (n=142), n (%)**	
Warfarin	74 (52.1)
Rivaroxaban	2 (1.4)
Dabigatran	45 (31.7)
Apixaban	21 (14.8)
If warfarin last INR (n=74), median (IQR)	2.3 (2.0 - 2.6)
**Antiplatelet (n=5), n (%)**	
ASA	2 (40.0)
Clopidogrel	2 (40.0)
Ticagrelor	1 (10.0)

INR: International Normalized Ratio

## Discussion

Atrial fibrillation (AF) is the most prevalent cardiac arrhythmia, significantly increasing the risk of thromboembolic complications compared to individuals without the condition [16]. Its clinical importance continues to grow due to the rising burden of non-communicable diseases (NCDs), particularly cardiovascular diseases (CVDs), in Kenya. The study assessed the demographic, clinical, treatment characteristics, and six-month outcomes post-hospital discharge using data from a single center.

The study population had a slightly higher proportion of females than males, with half of them aged 59. These demographic factors are crucial in AF prevalence and presentation. Age is a well-established risk factor for AF, with incidence increasing substantially with advancing age [17]. However, in many African settings, AF tends to manifest at a younger age than in high-income countries, primarily due to endemic risk factors such as hypertension, diabetes, and valvular disease, compounded by lifestyle and socioeconomic barriers to healthcare access. A similar study in Cameroon, which employed an identical methodology, reported comparable findings, with AF patients being younger and predominantly female [9]. Hypertension was the most prevalent comorbidity in the study cohort, consistent with epidemiological studies conducted in sub-Saharan Africa [5]. Among the identified risk factors, hypertension played a particularly prominent role, with studies consistently reporting that approximately 60% of AF patients have concomitant hypertension. This trend remains consistent across diverse populations, including African cohorts, highlighting the need for adequate blood pressure management as part of AF prevention strategies [18].

This study's predominant subtype was non-valvular AF, reflecting the increasing burden of NCDs. Less than a quarter of cases were attributed to rheumatic VHD, which aligns with findings from a 2010 study in Cameroon. That study reported that only a quarter of AF patients had VHD [9]. The rising prevalence of non-valvular AF in Africa is driven by epidemiological transitions marked by urbanization and lifestyle changes [19]. At discharge, all patients were prescribed rate control therapy despite fewer than a quarter having paroxysmal AF, with carvedilol being the most used beta-blocker. This finding aligns with a systematic review of AF in sub-Saharan Africa, which reported that over three-quarters of AF patients receive rate control therapy [5].

Stroke risk in non-valvular AF was assessed using the CHA_2_DS_2_-VASc score, initially validated by Gage *et al*. 2001 [16]. This tool was selected for its simplicity, reliability, and clinical utility. According to the CHA_2_DS_2_VASC score, patients with at least one stroke risk factor have an annual stroke risk of 2.8% and should be prescribed oral anticoagulation (OAC).

In the study, 77% of patients were on NOACs, with 67% meeting the criteria based on a CHA_2_DS_2_VASC score ≥ two or higher or having valvular AF. The remaining 10% were anticoagulated for other indications, such as pulmonary embolism. These findings align with global trends, where OAC use in AF patients has nearly doubled over the past decade, as reported in a systematic review and meta-analysis. Several factors have contributed to this increase, including introducing non-vitamin K antagonist oral anticoagulants (NOACs) and reshaping prescribing patterns, particularly among newly diagnosed patients and those previously ineligible for vitamin K antagonists (VKAs). Adopting the CHA_2_DS_2_-VASc score, alongside physician education initiatives, quality improvement programs, and pharmaceutical marketing, has further driven OAC uptake. Most study participants had low HAS-BLED scores, indicating a low bleeding risk.

Half of the study participants received warfarin, primarily for valvular AF, while the other half were prescribed NOACs or heparin, reflecting an increasing uptake of NOACs. This trend is consistent with an observational cohort study that examined OAC use before and after the introduction of direct oral anticoagulants (DOACs), showing that DOACs now account for approximately half of all anticoagulant prescriptions. Notably, rather than switching from VKAs to NOACs, the availability of DOACs has primarily expanded OAC use among previously untreated eligible AF patients. This suggests that NOACs are particularly favored for newly diagnosed cases and patients with contraindications or intolerance to VKAs [20]. The preference for NOACs over VKAs among freshly diagnosed AF patients aligns with European Society of Cardiology (ESC) AF guideline recommendations [21].

Encouragingly, anticoagulant use was maintained at the six-month follow-up. During this period, no cases of stroke or transient ischemic attack were reported, likely due to adherence to anticoagulation therapy. Additionally, there was only one participant with major bleeding complications, reinforcing the safety and efficacy of anticoagulation in the study cohort. At six months, approximately 80% of patients remained alive, with cardiac-related causes being the primary contributors to mortality among those who had passed away.

The study findings underscore the need for ongoing efforts to optimize AF management within the three leading national referral hospitals. Key priorities include ensuring sustained anticoagulation use and addressing modifiable risk factors such as hypertension. Further research is warranted to evaluate this population's long-term outcomes and barriers to anticoagulation adherence across other health facilities in Kenya, including public and private hospitals.

**Study limitations:** while this study offers valuable insights into the demographic, clinical, treatment characteristics, management, and outcomes post-discharge of atrial fibrillation (AF) in an African population, a few limitations are acknowledged. The study was designed to describe patient profiles rather than assess AF incidence.

The small sample size and six-month follow-up may not adequately capture long-term outcomes such as stroke, major bleeding, or anticoagulation adherence. The cause-of-death determination relied solely on clinical judgment, which may not be accurate without autopsy data. Follow-up challenges in low-resource settings, especially among rural patients, further limit data completeness. Additionally, the study focused on three leading national referral hospitals, which may restrict the generalizability of findings to other health settings. Nonetheless, substantial efforts were made to gather as much information as possible within these constraints.

Despite these limitations, this study provides baseline data on AF from three leading national referral hospitals in Kenya. It highlights the need for further research on long-term outcomes, multi-center collaborations, and strategies to enhance anticoagulation adherence and reduce AF-related morbidity and mortality across diverse settings.

## Conclusion

The study provided valuable insights into the clinical characteristics, management, and short-term outcomes of patients with atrial fibrillation (AF) in Kenya. The findings highlight that AF predominantly affects upper-middle-aged individuals, with hypertension emerging as the most prevalent comorbidity. Future research on multi-center collaborations, long-term outcome assessments, and strategies to overcome barriers to AF care in resource-limited settings is needed.

### What is known about this topic

- Atrial fibrillation (AF) is the most common sustained cardiac arrhythmia. It is characterized by disorganized electrical activity in the atria, which leads to ineffective atrial contractions and an irregular ventricular response;
- Evidence suggests that African populations with AF often present with more severe clinical manifestations compared to patients in high-income countries.

### What this study adds

- Atrial fibrillation in Kenya affects the younger population with a high proportion of hypertension;
- Anticoagulation adherence was strong among the participants; however, mortality remains high.
